# Transcriptome analysis of mRNA and miRNA in skeletal muscle indicates an important network for differential Residual Feed Intake in pigs

**DOI:** 10.1038/srep11953

**Published:** 2015-07-07

**Authors:** Lu Jing, Ye Hou, Hui Wu, Yuanxin Miao, Xinyun Li, Jianhua Cao, John Michael Brameld, Tim Parr, Shuhong Zhao

**Affiliations:** 1 Key Lab of Agricultural Animal Genetics and Breeding, Ministry of Education, College of Animal Science and Veterinary Medicine, Huazhong Agricultural University, Wuhan, 430070, P. R. China; 2 Division of Nutritional Sciences, University of Nottingham, School of Biosciences, Loughborough, Leics. LE12 5RD, United Kingdom

## Abstract

Feed efficiency (FE) can be measured by feed conversion ratio (FCR) or residual feed intake (RFI). In this study, we measured the FE related phenotypes of 236 castrated purebred Yorkshire boars, and selected 10 extreme individuals with high and low RFI for transcriptome analysis. We used RNA-seq analyses to determine the differential expression of genes and miRNAs in skeletal muscle. There were 99 differentially expressed genes identified (q ≤ 0.05). The down-regulated genes were mainly involved in mitochondrial energy metabolism, including *FABP3, RCAN, PPARGC1 (PGC-1A), HK2* and *PRKAG2*. The up-regulated genes were mainly involved in skeletal muscle differentiation and proliferation, including *IGF2*, *PDE7A, CEBPD, PIK3R1* and *MYH6*. Moreover, 15 differentially expressed miRNAs (|log2FC| ≥ 1, total reads count ≥ 20, p ≤ 0.05) were identified. Among them, miR-136, miR-30e-5p, miR-1, miR-208b, miR-199a, miR-101 and miR-29c were up-regulated, while miR-215, miR-365-5p, miR-486, miR-1271, miR-145, miR-99b, miR-191 and miR-10b were down-regulated in low RFI pigs. We conclude that decreasing mitochondrial energy metabolism, possibly through *AMPK* - *PGC-1A* pathways, and increasing muscle growth, through *IGF-1/2* and *TGF-β* signaling pathways, are potential strategies for the improvement of FE in pigs (and possibly other livestock). This study provides new insights into the molecular mechanisms that determine RFI and FE in pigs.

Feed accounts for more than 60% of the costs for pig production, therefore improving feed efficiency (FE) is one of the major ways to reduce costs in the pig industry. FE can be measured as feed conversion ratio (FCR) or residual feed intake (RFI)^1^. FCR is the feed intake divided by the weight gained during a specified period. RFI is defined as the difference between the actual and the predicted dry matter (DM) intakes of each animal, based on its metabolic body weight and average weight gain during a specified period^2^. Thus, animals with higher RFI/FCR are less efficient at converting feed into body mass, whereas those with lower RFI/FCR are more efficient. Previous studies indicated that the heritability of RFI is 0.14–0.40 and FCR is 0.13–0.31^3^,^4^,^5^, and a strong correlation exists between them (R equals 0.76–0.99)^3^.

With microsatellite typing based QTL mapping, Zhang and his colleagues identified three genomic regions on SSC2, SSC7 and SSC9 in a White Duroc × Chinese Erhualian F2 segregated population^6^, associated with the feed consumption and feeding behavior traits, average daily feed intake (ADFI), feed conversion ratio (FCR), number of visits to the feeder per day (NVD) and average feeding rate (AFR). Recently, a whole genome association analysis study showed that SNPs located on SSC7, SSC13, SSC14 and SSC17 were significantly associated with the RFI trait in a Yorkshire pig population selected for high and low RFI^7^. Furthermore, 10 SNPs identified using high-density SNP chip analysis^8^ had significant association with FCR in a Duroc pig population, 2 of them were on SSC4 and the others were on SSC 14. However, by comparing chromosome regions and genes related with FE, it is hard to find a single region or one major candidate gene. Hence, the candidate genes relating to FE in pigs are not well understood.

Three biological processes have been reported to be associated with FE in pigs through microarray transcriptome analysis, including glucose metabolism, lipid metabolism and muscle development (myogenesis). Gene expression profiling in liver and adipose tissue following acute caloric restriction of pigs, suggested that lipid metabolism, mitochondrial activity and glucose synthesis were all related with FE^9^. Moreover, lipogenic and steroidogenic genes were down-regulated in both liver and adipose tissue of Yorkshire gilts with lower RFI^10^. In cattle, 161 genes were differentially expressed between animals with high and low RFI. These genes were related with several gene networks, including cell growth and differentiation, lipid metabolism and carbohydrate metabolism^11^. No major gene has been identified to regulate FE in pigs^12^.

MicroRNAs (miRNAs) are small noncoding RNAs of 18 to 23 nucleotides, which play important roles as post-transcriptional regulators^13^. miRNAs have also been reported to be associated with feed efficiency and energy metabolism. In cattle, the distribution of SNPs in miRNA motifs associated with RFI was much more significant compared with SNPs in other regions^14^. In addition, one SNP of the stearoyl-CoA desaturase (SCD) gene, within a predicted target site for 2 miRNAs (ssc-miR-185 and ssc-miR-491), was significantly associated with daily body weight gain and FCR in cattle^15^. Besides, there are some differentially expressed miRNAs in fish with different growth rates, with let-7j, miR-140, miR-192, miR-204, miR-218a, miR-218b, miR-301c and miR-460 all being down regulated in fast-growing fish. Moreover, let-7b, let-7c, miR-133, miR-152, miR-15a, miR-193a, miR-30b and miR-34 were all up regulated in fast-growing fish^16^. In March 2015, Li and his colleagues presented the first systematic identification and characterization of lincRNAs in fetal porcine skeletal muscle, which identified 570 porcine lincRNAs, but most were related to skeletal muscle development^17^. However, to our knowledge there are as yet no studies relating porcine FE and miRNA expression.

In this study, we used mRNA and miRNA sequencing to profile the skeletal muscle transcriptome and thereby identify genes and miRNAs that were differentially expressed between pigs with different feed efficiencies. We identify a number of genes and miRNAs that were significantly differentially expressed between high and low RFI pigs. Importantly, mitochondrial energy metabolism regulatory pathways (e.g. PGC-1a and *AMPK*) were down-regulated and muscle growth regulatory pathways (e.g. *IGF-1/2* and *TGF-β*) were upregulated in skeletal muscle from the more efficient (low RFI) pigs.

## Results

### Animal performance

An original 238 castrated purebred Yorkshire boars were grown from 30 to 90 kg (average body weight) on a commercial pig feed (see Materials and Methods). Daily feed intake (DFI) and average daily gain (ADG) were measured and the FCR and RFI determined. From this data, two sub-groups were selected with Low or High RFI (RFI_L and RFI_H respectively). Importantly, the total weight gained and the time taken to do so was not significantly different between the two groups. The High RFI group (RFI_H) had RFI and FCR values of 0.43 ± 0.19 (Kg/day) and 3.07 ± 0.15 respectively, compared with −0.28 ± 0.07 (Kg/day) and 2.19 ± 0.06 for the more efficient, Low RFI group (RFI_L, p < 0.05 for both RFI and FCR, Table 1). The lower FCR seen in the RFI_L group was achieved by a reduced DFI (p = 0.02) and, interestingly, a trend for an increase in ADG (p = 0.08) compared with the RFI_H group (Table 1). Although the average metabolic body weight gain (AMBW) was higher in the RFI_L group, it was not statistically significant (p = 0.65). Two assessments of body fat, average back fat (ABF) thickness and intramuscular fat (IMF) content, showed no significant differences, suggesting that changes in RFI or FCR were not mediated by significant changes in body fat.

### RNA sequencing data mapping and annotation

A total of 6 cDNA libraries were sequenced from the longissimus dorsi muscle of RFI_L and RFI_H groups (n = 3 from each). The RNA sequence reads have been submitted to the NCBI Gene Expression Omnibus under accession E-MTAB-2946. After removing the adaptors and filtering, RNA-seq yielded from 7.8 M to 9.6 M single-end reads for all 6 samples, more than 99.99% reads were qualified (Supplementary Table S1).

After mapping clean reads to the porcine genome, 89.03%–91.11% reads were successfully aligned, with 51.5–58.4% of reads mapped to CDS regions, 0.53–0.59% of reads mapped to 5’UTR regions, 12.36–14.37% of reads mapped to 3’UTR regions and 10.14–12.66% of reads mapped to introns or intergenic regions, while 8.83–11.00% of reads had multiple alignments (Supplementary Figure S1a). The reads distribution in each chromosome were analyzed according to the porcine genome, and about 10.19–21.29% of the total reads mapped to the mitochondrial genome, which was higher than the proportion of reads mapped to other chromosomes (Supplementary Figure S1b). The mitochondrial genome distribution of the RFI_H group was 19.73 ± 4.98%, compared with 14.33 ± 3.06% in the RFI_L group (Supplementary Figure S1c). This indicates that genes encoded for by the mitochondrial DNA account for a large proportion of the genes differentially expressed in skeletal muscle.

### miRNA sequencing data mapping and annotation

A total of 2 cDNA libraries were sequenced from the longissimus dorsi muscle of RFI_L and RFI_H groups (1 pool of n = 5 for each group), each cDNA library included the 3 samples used for RNA sequencing). After sequencing, a total of 13,307,888 reads were obtained from the RFI_H group and 11,744,483 reads from the RFI_L group. The miRNA sequence reads have been submitted to the NCBI Gene Expression Omnibus under accession E-MTAB-2949. After removing reads with non-canonical letters or with low quality, the 3’ adapter was trimmed and the sequences shorter than 18 nt discarded. In total, 12,503,358 (RFI_H), and 10,408,712 (RFI_L) clean reads were obtained, which corresponded to 93.95% and 88.63% respectively of the raw reads from each small RNA library. The length distribution of clean reads showed that most of the reads were between 21–23 nt in length, and read counts with 22 nt were highest (Supplementary Figure S2a).

A total of 402 mature miRNAs were identified. Among them, 152 were annotated porcine miRNAs already present in miRbase v20, 153 were miRNAs homologous to human or mouse, while 97 were novel miRNAs not homologous to any other species (Supplementary Figure S2b). The distribution of miRNAs on each chromosome depended on the number of annotated miRNAs on each chromosome (Supplementary Figure S2c).

### Differentially expressed genes between low and high RFI pigs

In the present RNA-seq study, 30,484 genes were detected in the skeletal muscle of all 6 individuals. A total of 645 genes were differentially expressed, with a criteria of at least a 2 fold difference and a p-value less than 0.05 (|log2FC| ≥ 1, p < 0.05), of which 99 genes had a q-value ≤ 0.05. Of the 99 differentially expressed genes, 60 genes were well annotated on the *Sus Scrofa* genome (version Sscrofa10.2.72), with 45 genes being up-regulated and 54 genes down-regulated in the low-RFI group (Supplementary Table S2). Table 2 shows the top 20 differentially expressed (DE) genes, the top 10 genes with either higher or lower expression in low RFI compared to high RFI pigs.

**Figure 1:**
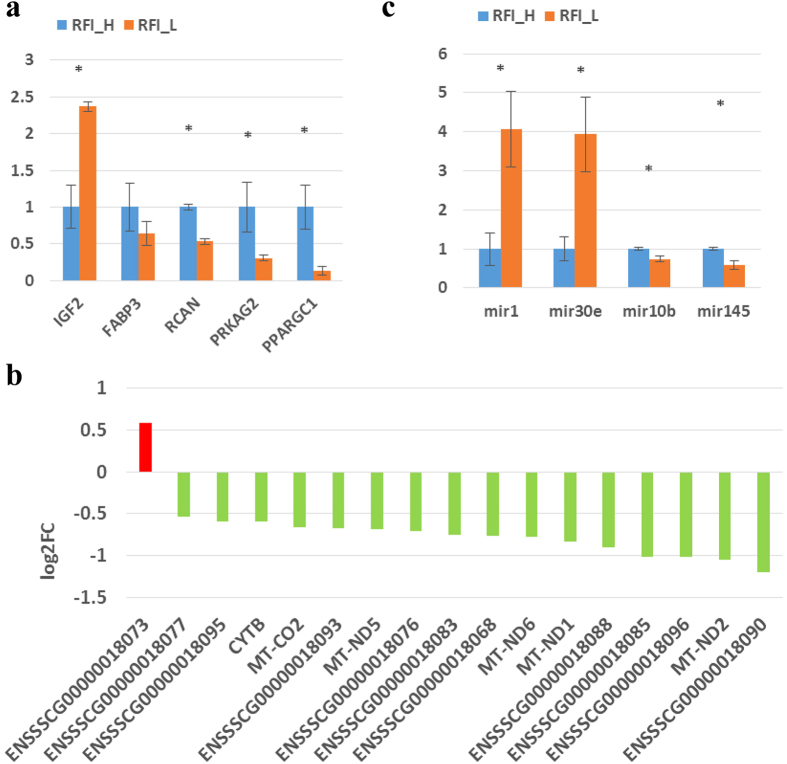
Validation of differentially expressed genes in LD muscles from high and low RFI pigs. (**a**) qPCR results for *IGF2, FABP3, RCAN, PRKAG2* and *PPARGC1I* genes, analyzed by the ∆∆Ct method. * significant difference between RFI_H and RFI_L pigs. (**b**) The log_2_FC expression levels of mitochondrial DNA encoded genes. (**c**) qPCR results for mir1, mir30e, mir10b and mir145, analyzed by the ∆∆Ct method. * * significant difference between RFI_H and RFI_L pigs.

To validate the differential expression of genes, eight genes were selected for qRT-PCR analysis. Compared with the RFI_H group, expression of *FABP3, RCAN, PPARGC1,* and *PRKAG2 mRNA* were all lower in RFI_L muscles, whereas expression of *IGF2* mRNA was higher in RFI_L muscles (Fig. 1a). Expression of 4 of the selected genes (*IGF2, RCAN, PPARGC1* and *PRKAG2*) showed significant differences between the RFI_H and RFI_L groups. Hence the qRT-PCR analyses largely confirmed the RNA-seq data, with the correlation coefficient of the fold-change (FC) values from the two methods being 0.99 and the R^2^ for the linear regression also being 0.99, indicating the reliability of the RNA-seq analysis.

In addition, we analyzed the expression levels of various mitochondrial genes. There were 17 mitochondrial coding genes detected (log2FC ≥ 0.5) and 16 genes (94.1%) were down regulated in the skeletal muscle from low RFI pigs (Fig. 1b, Supplementary Table S3).

### Differentially expressed miRNAs between low and high RFI pigs

In the miRNA-sequencing study, 25 miRNAs were differentially expressed with a criteria of at least a 1.5 fold difference and total reads count no less than 20 (|logFC| ≥ 1, total ≥ 20, p ≤ 0.05). Of these 25 miRNAs, 14 were up-regulated and 11 down-regulated in RFI_L pigs (Table 3). Six of them were not found in the *Sus scrofa* miRNA database (miRbase v20), but are homologous with human. Six novel miRNAs were identified as being differentially expressed; the secondary structures of which were predicted by mirDeep2 (Supplementary Figure S3) and the predicted scores from mirDeep2 listed in Supplementary Table S4.

To validate the differential expression identified by the miRNA sequencing, miR-1, miR-30e, miR-10b and miR-145 were selected for qRT-PCR analysis. Compared to RFI_H muscles, expression of miR-1 and miR-30e miRNAs was higher, whereas expression of miR-10b, and miR-145 was lower in RFI_L muscles (Fig. 1c). All the selected miRNAs showed significant differences between RFI_H and RFI_L groups, confirming the results of miRNA-seq, and the correlation coefficient for the fold-change values (FC) from the two methods was 0.99 and the R^2^ for linear regression was also 0.99, indicating the reliability of the miRNA-seq analysis. The differentially expressed miRNAs were functionally involved in energy metabolism processes and pathways, which were all down regulated in low RFI pigs.

### Pathway analysis of differentially expressed genes

For gene ontology (GO) biological processes analysis, 54 well annotated genes among the 99 significant DE genes were submitted and 45 of these genes were available in DAVID v6.7.

A total of 26 GO terms were enriched (Fisher Exact Probability Test, p < 0.05), which can be divided into 5 major groups: 1) Genes relating to general metabolism, 2) Genes relating to energy metabolism, 3) Genes relating to lipid metabolism, 4) Genes relating to cell differentiation, and 5) Genes relating to biosynthetic processes (Table 4).

Most genes related to energy metabolism, cell differentiation and biosynthesis were down regulated in pigs with low RFI. For example *PPARGC1, PRKAG2, HK2* are related to energy metabolism; NOR-1, RCAN, ESRRB, ESRRG and JUNB are related to cell differentiation; and LIPG and GM2A are related to lipid catabolism. The significant down regulation (p ≤ 0.05) of all those genes indicates that low RFI pigs may be more efficient due to (i) a decrease in energy expenditure because of an inhibition of metabolic processes and/or (ii) an increase in skeletal muscle growth.

### Pathway analysis of differentially expressed miRNAs

To better understand the biological functions of the 25 differentially expressed miRNAs identified, we predicted the potential target genes of these miRNAs. There were 12,687 terms of target genes returned, including 6,744 unique genes (Supplementary Table S5). Through the miRNA-targeted pathway union analysis, we found 55 KEGG pathways significantly (Fisher Exact Probability Test, p < 0.05) related with genes targeted by up or down-regulated miRNAs (Supplementary Table S6). A lot of pathways were involved in energy metabolism and skeletal muscle growth, including the *TGF-beta* signaling pathway, PI3K-Akt signaling pathway, mTOR signaling pathway, GnRH signaling pathway and Hypertrophic cardiomyopathy (HCM).

**Figure 2:**
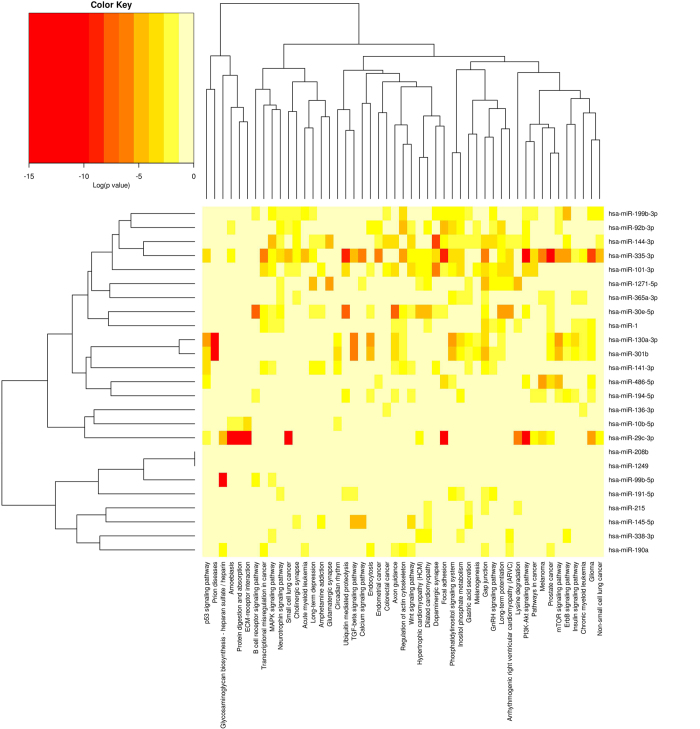
Heat map and Cluster patterns of the differentially expressed miRNAs and target gene related pathways. Heat map of miRNAs versus pathways, miRNAs are clustered together by exhibiting similar pathway targeting patterns, and pathways are clustered together by related miRNAs. As porcine genes were not included in the current version of DIANA miRPath, prediction was performed using human miRNAs.

To further classify and predict the function of differentially expressed miRNAs, we also performed hierarchical clustering of differentially expressed miRNAs and their target pathways (Fig. 2). Some miRNAs with the same regulation pattern or similar function were clustered together, for example miR-130a-3p and miR-301b-3p were clustered together. After checking the mature sequences of these two miRNAs, we found they have exactly the same seed sequences and both of them are in the same miRNA family.

### The mRNA – miRNA Regulatory network analysis

We investigated the miRNA-gene interactions between 54 well annotated differentially expressed genes and 25 differentially expressed miRNAs. There were 6 significantly enriched pathways (Fisher Exact Probability Test, p < 0.05, Supplementary Table S7), including Adipocytokine signaling pathway, Insulin signaling pathway, Hypertrophic cardiomyopathy (HCM), Bacterial invasion of epithelial cells, Viral carcinogenesis, and Phagosome.

The genes and miRNAs related to those KEGG pathways are listed in Table 5. These pathways were mainly related to energy metabolism and skeletal muscle growth, and include PPARGC1A, PIK3R1, PRKAG2, hsa-miR-130a-3p, hsa-miR-30e-5p, and hsa-miR-335-3p.

### A key network of mRNA and miRNA in pig muscle potentially regulates RFI in pigs

**Figure 3:**
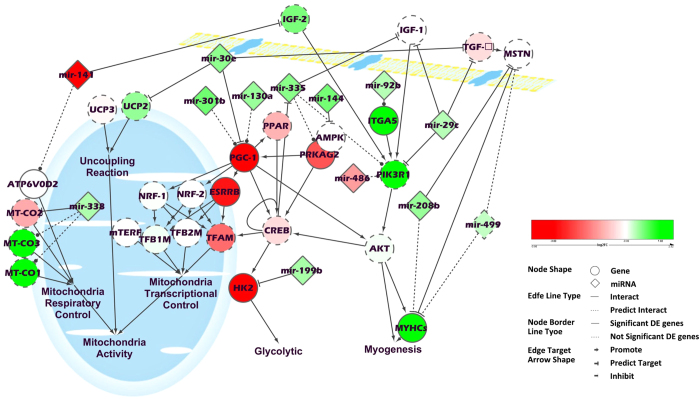
The key network of genes and miRNAs found to be differentially expressed in skeletal muscle from low RFI compared with high RFI pigs. The network diagram was made using Cytoscape.

Cytoscape v3.0.1 was used to integrate a potential network of differentially expressed genes and miRNAs interacting in pig skeletal muscle that might lead to differences in RFI (Fig. 3). When looking into the differentially expressed genes, we found that most of those genes were involved in mitochondrial activity, glycolysis or myogenesis pathways and were actually connected directly or indirectly through just one or two genes. In this network, the mitochondrial activity was separated into 3 parts: the uncoupling reaction, the mitochondria respiratory control and the mitochondria transcriptional control.

In mitochondrial activity, the expression levels of *PGC-1, ESRRB* and *TFAM* were down-regulated in the low RFI pigs more than other genes, which suggests that the down-regulation of mitochondrial activity might possibly be through mitochondria transcriptional control.

In the glycolysis pathway, the upstream genes of *CREB* were also involved in mitochondria transcriptional control. Most of these genes were down-regulated in low RFI pigs, such as *PGC1, PPAR* and *PRKAG2. HK2*, the gene downstream of *CREB*, was also down-regulated in low RFI pigs. *HK2* is a hexokinase that phosphorylates glucose to produce glucose 6-phosphate^18^. Hexokinase regulates glycolysis as the first rate-limiting enzyme^19^. Moreover, the miRNAs, which were reported or predicted to target and therefore inhibit these genes, were all up-regulated in low RFI pigs, including mir-30e, mir-301b, mir-130a, mir-335 and mir-199b. This suggests an overall decrease in glycolysis and mitochondrial activity in skeletal muscle of low RFI pigs.

In contrast, most genes involved in the myogenesis pathway were up-regulated in low RFI pigs, such as *IGF-2*, *ITGA5*, *PIK3R1* and *MYHC*s. Also, *TGF-β*, which is an inhibitor of myogenesis, was down-regulated in low RFI pigs. Interestingly, mir-141, which targets *IGF-2,* was down-regulated and mir-30e, which targets *TGF*-β, was upregulated in low RFI pigs. These results indicate that the increased efficiency of low RFI pigs is associated with enhanced skeletal muscle growth.

## Discussion

Two groups of pigs were identified which had either high or low residual feed intake. Those pigs in the RFI_L group were more efficient, having a lower FCR than the RFI_H group, due to lower DFI and slightly higher ADG. A common observation in these types of study is that selection for greater feed efficiency often targets reduced feed intake, which has been reported as undesirable as it can reduce growth and reproductive performance^20^,^21^. However in this study there was trend for a positive effect on ADG in the RFI_L pigs, despite a reduction in feed intake. From the estimates of fat deposition, there were no changes in body fat, although the values for back fat and IMF tended to be lower in the RFI_L pigs. In addition the AMBW of the RFI_L was higher than RFI_H, but this was not statistically significant. When combined with the assessment of body fat, this suggests an increase in lean tissue growth and a corresponding decrease in fat in the more efficient RFI_L pigs.

We identified 99 differentially expressed genes and 25 differentially expressed miRNAs in LD muscle from pigs with significantly different RFI. These genes and miRNAs corresponded to two key pathways/functions, one related to mitochondria and energy metabolism and the other related to skeletal muscle growth. The energy metabolism and growth of skeletal muscle may be the two key factors responsible for low RFI and therefore increased efficiency of pigs.

Mitochondria produce more than 90% of cellular energy through oxidative phosphorylation (OXPHOS), but also waste energy and generate heat through uncoupling reactions^22^. In our study, both the uncoupling reactions and OXPHOS were down-regulated in low RFI pigs. Uncoupling proteins (UCP) play a critical role in energy-dissipating uncoupling reactions through [H+] leakage in mitochondria. It has been reported that overexpression of *UCP3* in skeletal muscle increases energy expenditure and decreases feed efficiency in mice^23^,^24^. In our study, we found *UCP2*, the ubiquitously expressed isoform of *UCP*, was down regulated in muscle from low RFI pigs. Also miR-30e, which directly targets *UCP2,* was up regulated in RFI_L pigs. We also found that genes associated with OXPHOS and ATP synthesis and mitochondria transcriptional control were all down-regulated in low RFI pigs, including *COX*-*I*, *COX-III*, *COX-IV*, *COX-V*, *PGC-1*, *PRKAG2*, *ESRRGB*, and *HK2*. MiR-338 has been confirmed to inhibit *COXIV* at mRNA and protein levels^25^ and miR-338 was up-regulated in low RFI pigs.

*PGC-1*, also called *PPARGC1* or *PPARGC1A*, plays an important role in mitochondrial biogenesis, by activating cAMP response element binding protein (*CREB*) and nuclear respiratory factors (NRFs). These nuclear factors then increase the transcription of mitochondrial transcription factors (*TFAM*, *TFB1M*, *TFB2M*) which promote mitochondrial biogenesis^26^. Overexpression of *PGC-1* has been reported to increase energy expenditure and mitochondrial number^27^. In our study, *PGC-1* and all these down-stream genes related to mitochondrial biogenesis were down-regulated in the low RFI pigs. Also, we found that expression of almost all the genes located in the mitochondrial DNA were down-regulated in low RFI pigs, suggesting either a reduction in the number of mitochondria or a general down-regulation of mitochondrial function. In addition, *CREB* has been reported to be targeted by miR-335, which was up-regulated in low RFI pigs^28^. Therefore, these results indicate that mitochondrial biogenesis/function and energy expenditure were reduced in low RFI pigs.

*AMPK* is a key regulator of cellular and whole-body energy balance, which is activated by an increase in the AMP/ATP ratio^29^,^30^. *AMPK* can also increase mitochondrial proteins of oxidative metabolism, as well as promote the expression of Hexokinase II (*HK2*) through *CREB* in skeletal muscle^31^. In our study, we found that HK2 and Protein kinase, AMP-activated, gamma 2 non-catalytic subunit (*PRKAG2*), which is a member of *AMPK* gamma subunit family, were both down regulated in low RFI pigs. Also, miR-144, which has been reported to inhibit the phosphorylation of *AMPK* alpha (*AMPKα*), was increased in low RFI pigs^32^. These results indicate that the level of energy metabolism in skeletal muscle was probably reduced in low RFI pigs compared to high RFI pigs.

Further KEGG pathway analyses indicated that *PGC-1* and *PRKAG2* are involved in the adipocytokine signaling pathway (KEGG hsa04920). In this pathway, mitochondrial fatty acid β-oxidation is increased by the expression of *PGC-1,* while *PRKAG2* increases appetite by up-regulating AGRP and NPY. Recently, Lindholm-Perry found the expression level *PRKAG2* in the rumen was associated with ADFI in cattle^33^, although the direction of the association depended upon season. Thus, *PGC-1* and *PRKAG2* are potentially key genes in the molecular regulation of feed efficiency. Although this pathway is termed the “adipocytokine signaling pathway”, the genes are expressed in lots of tissues/ cell types where they have similar functions.

As a *PPARα* coactivator, *PGC-1* plays a key role in the transcriptional control of genes encoding mitochondrial fatty acid β-oxidation (FAO)^34^. It is reported that *PPARα/PGC-1* signaling pathway can be activated by chronic overnutrition and obesity, resulting in the up regulation of fatty acid β-oxidation related genes, such as *FATP1, FACS1, UCP2* and *UCP3*^35^. *PRKAG2* also plays a critical role in regulating cellular fatty acid metabolic pathways^36^. Thus, the down regulation of these genes indicate that mitochondrial fatty acid β-oxidation was reduced in low RFI pigs compared to high RFI pigs. Hence we propose that the pathway is more to do with regulating whole body energy balance via effects on energy expenditure or appetite, rather than relating to adipose tissue metabolism *per se*.

A number of miRNAs related to skeletal muscle growth and development were differentially expressed between low and high RFI pigs, including miR-208b, miR-499, miR-29c, miR-1 and miR-99b. The transforming growth factor-beta (*TGF-β*) signalling pathway, which includes Myostatin (*MSTN*), is considered the most potent negative regulator of skeletal muscle growth and development^37^. It has been reported that, miR-29 and miR-30b are both inhibitors of *TGF-beta*^38^,^39^,^40^, while miR-208b and miR-499 inhibit the *MSTN* gene^41^. Also, overexpression of miR-29 promotes myogenic differentiation in C2C12 cells, due to the reduction in *TGF-beta*, which inhibits differentiation^42^. Furthermore, miR-99b has been reported to be directly stimulated by the *TGF-β* signaling pathway^43^ and miR-99b is reported to inhibit *IGF-1*/mTOR signaling by targeting AKT1, IGF1R and mTOR^44^,^45^.

In the present study, we found miR-29c, miR-30b, miR-208b and miR-499 were all up-regulated in low RFI pigs, while miR-99b was down-regulated. All these results suggest that muscle growth and development was increased in low RFI pigs via the inhibition of the *TGF-β* signaling pathway and stimulation of the *IGF-1*/mTOR signaling pathway. We also found *IGF-2* and *MYHC*s were up-regulated in low RFI pigs. It appears from the pig growth performance data that these changes were associated with a slightly increased ADG, even though DFI was reduced, suggesting that the RFI_L pigs were able to sustain their growth whilst reducing their feed intake.

Thus, based on the entire expression profiles of both mRNAs and miRNAs, we conclude that the improvements in feed efficiency in low RFI pigs are due to inhibition of skeletal muscle mitochondrial activity through *PGC-1/TFAM* and *PRKAG2* combined with the stimulation of muscle growth through *TGF-β* and *IGF-1/2* signaling pathways.

## Conclusions

Overall, we identified 99 mRNAs and 25 miRNAs that were differentially expressed in skeletal muscle from pigs with different RFI. These genes were functionally related to metabolism, particularly energy and lipid metabolism, as well as biosynthetic processes and muscle cell growth and differentiation. A number of genes involved in energy metabolism were down-regulated, whereas quite a few miRNAs that target energy metabolism genes were up-regulated in muscle from low RFI pigs. Similarly, a number of genes and miRNAs which stimulate skeletal muscle differentiation and proliferation were up-regulated. We propose that feed efficiency in pigs can be improved by reducing energy metabolism in muscle, particularly mitochondrial metabolism, and/or by enhancing skeletal muscle growth and we identify a number of miRNAs and genes that might be targets for manipulation. This study enhances our understanding of molecular mechanisms regulating feed efficiency in pigs.

## Materials and Methods

### Animals and tissues

In this study, 238 castrated purebred Yorkshire boars were grown from 30 to 90 Kg, the average period of study was 67.35 days. Pigs were slaughtered at 90 kg according to a standard procedure approved by Biological Studies Animal Care and Use Committee^46^, Hubei Province, P. R. China. They were individually fed *ad libitum* a complete mixed commercial feedlot ration (see Supplementary Table S8), using ACEMA 64 automated individual feeding systems in the Agricultural Ministry Breeding Swine Quality Supervision Inspecting and Testing Center (Wuhan). All the methods in this study were carried out in accordance with the approved guidelines from Regulation of the Standing Committee of Hubei People’s Congress . All experimental protocols were approved by the Ethics Committee of Huazhong Agricultural University.

For RNA sequencing, the 3 pigs with the highest RFI (named RFI_H group) and the 3 pigs with lowest RFI (named RFI_L group) were selected from the 238 pigs, each RFI group having no difference in starting body weight (Table 1). For miRNA sequencing, the 5 pigs with the highest RFI (named RFI_H group) and the 5 pigs with the lowest RFI (named RFI_L group) were selected.

Within 30 minutes after slaughter, a piece of longissimus dorsi muscle of each animal was sampled at the thoracolumbar junction. All tissue samples were immediately frozen in liquid nitrogen and stored at −80 °C for RNA isolation.

### Phenotypes

RFI was calculated by a linear regression model according to the records of daily feed intake (DFI), average daily gain (ADG) and mid-test metabolic body weight (MBW) of all the pigs.

The base model used was Y_j_ = β_0 _+ β_1_MBW_j_ + β_2_ADG_j_ + e_j_ , where Y_j_ is the DMI of the j^th^ animal, β_0_ is the regression intercept, β_1_ is the regression coefficient on MBW, β_2_ is the regression coefficient on ADG, and e_j_ is the uncontrolled error of the j^th^ animal.

### RNA preparation and sequencing

Total RNA was extracted with Trizol reagent (Invitrogen, USA), according to the manufacturer’s instructions. RNA sequencing libraries were prepared for each RNA-seq sample using “TruSeq® Stranded Total RNA Sample Preparation kit (Illumina®)” and all of the procedures and standards were performed according to the manual supplied with this kit. For miRNA sequencing, the RNA samples from the 5 different pigs in the same group were pooled together based on an equal RNA quantity. TruSeq® Small RNA Sample Prep Kit (Illumina®) was used for miRNA sequencing library preparation and all of the procedures and standards were performed according to the manual supplied with this kit. After quality control, sequencing of all the libraries was performed by HiSeq2000. Solexa sequencing was performed at the Beijing Genomics Institute (BGI), Beijing, China.

### Analyses of RNA-Seq data

Having transferred the RNA-Seq results from Illumina fastq format to standard Sanger fastq format with fq_all2std.pl, data were processed with the Tophat-Cufflinks pipeline^47^. The porcine reference genome and gtf annotation file were downloaded from Ensembl (Sscrofa10.2.72) and build index with bowtie version 2.1.0. TOPHAT (version 2.0.9) was used to align reads to the genome with the option --library-type fr-firststrand. Cufflinks (version 2.1.1) was used for transcriptome assembling, and Cuffdiff script from Cufflinks was used for gene expression analysis with the option -classic-fpkm. The expression level of each gene was represented by the FPKM value, which means fragments per kilobase of exon per million fragments mapped, and was calculated by the following formula^48^:
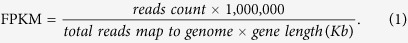
Finally, q ≤ 0.05 was set as the threshold for differentially expressed (DE) gene selection.

### Analyses of miRNA-Seq data

Firstly, miRNA-Seq data were transferred from Illumina fastq format to fasta format and then the miRNA-seq datasets analyzed with mirDeep (v2.0.0.5)^49^,^50^. The porcine genome (Sscrofa10.2.72) was downloaded from Ensembl, and the miRNA reference was obtained from the miRBase database (version 20)^51^,^52^,^53^,^54^,^55^.

The miRNA expression level of each library was normalized by the following formula^56^:


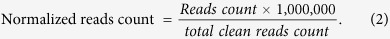


The P-value between the two libraries was calculated using the following formulas^57^:


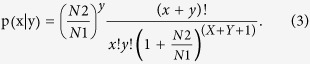


N1 and N2 represent the total numbers of clean reads in the two small RNA libraries. The q-value was calculated by fdrtool in R^58^. Finally, |log2FC| ≥ 1, total reads count ≥20, p ≤ 0.05 was set as the threshold for selection of differentially expressed (DE) miRNA.

### Q-RT-PCR validation of differentially expressed genes and miRNAs

Relative expression levels of the differentially expressed genes and miRNAs in muscle were quantified by real-time PCR. The RPL4 gene and 18S RNA were selected as the internal controls for qRT-PCR validation because of their stable expression in skeletal muscle tissues. The poly(A)-tailed RT-PCR method^59^ was performed for miRNA reverse transcription. Primer sequences and PCR conditions for analyzed genes and miRNAs are listed in Supplementary Table S9. The reactions were performed on a Roche Lightcycler 480 Sequence Detection System using SYBR Green PCR Master Mix (TOYOBO, QPK201), following the instruction manual. The 50 μL reaction mixture consisted of 5 μL cDNA, 25 μL 2 × SYBR Green PCR Master Mixture, 0.5 μM each primers and water. The qPCR profiles began with initial denaturation at 95 °C for 10 min, and then followed by 40 cycles of 95 °C denaturation for 15s, annealed at 60 °C for 15s, and 72 °C for 15s extension. A dissociation curve was generated at the end of the last cycle by collecting the fluorescence data from 58 °C to 95 °C. The 2-ΔΔ Ct method was employed for relative gene expression level analysis. For each gene, the average ΔCt value of the RFI_L group was used as reference to calculate the ΔΔCt value, and Student’s t-test was used to analyze the expression difference between the 2 groups^59^.

### Potential target gene prediction of miRNAs

To explore the potential function of miRNAs with significant differential expression in the two groups, potential target genes and pathways of miRNAs were predicted by DIANA miRPath (v.2.0)^60^. As porcine genes were not included in the current version of DIANA miRPath, prediction was performed using human miRNAs. The P-value threshold was 0.05 and MicroT threshold was 0.8^60^ (http://www.microrna.gr/miRPathv2).

### Gene ontology and pathway analyses

The human homologous Ensembl Gene IDs of the identified DE genes and miRNA target genes were utilized for the following bioinformatics analysis. Gene enrichment in gene ontology (GO) biological processes and pathways were performed with the DAVID Bioinformatics Resources v6.7 (http://david.abcc.ncifcrf.gov/)^61^,^62^. Cutoff criteria were EASE scores less than 0.01 (GO enrichment) or 0.05 (Gene enrichment in pathways). EASE score were given by DAVID, which is a modified Fisher’s exact test. Cytoscape (v3.0.1) was used to create the potential important network(s) of DE genes and miRNAs^63^.

## Additional Information

**How to cite this article**: Jing, L. *et al.* Transcriptome analysis of mRNA and miRNA in skeletal muscle indicates an important network for differential Residual Feed Intake in pigs. *Sci. Rep.* **5**, 11953; doi: 10.1038/srep11953 (2015).

## Supplementary Material

Supplementary Information

Supplementary Table S1

Supplementary Table S2

Supplementary Table S3

Supplementary Table S4

Supplementary Table S5

Supplementary Table S6

Supplementary Table S7

Supplementary Table S8

Supplementary Table S9

## Figures and Tables

**Table 1:** Animal performance of Yorkshire pigs used in RNA and miRNA sequencing.

	RFI_H	RFI_L	p-value
n	5	5	
FCR	3.07 ± 0.15	2.19 ± 0.06	4.25E-06
RFI (kg/day)	0.43 ± 0.19	−0.28 ± 0.07	1.13E-04
Testing Days	59 ± 6.84	55 ± 9.55	0.52
DFI	2.62 ± 0.3	2.11 ± 0.21	0.02
ADG	0.85 ± 0.07	0.96 ± 0.09	0.08
Initial BW (kg)	40.42 ± 4.2	40.54 ± 6.15	0.98
Final BW (Kg)	90.36 ± 2.36	92.76 ± 3.45	0.28
TBG	22.88 ± 0.55	23.2 ± 1.21	0.53
AMBW	22.88 ± 0.55	23.2 ± 1.21	0.65
ABF(mm)	19.84 ± 3.43	20.74 ± 3.4	0.72
LMA (cm^2^)	46.86 ± 6.14	47.34 ± 1.25	0.88
IMF	2.38 ± 0.0082%	2.18 ± 0.0057%	0.70

DFI - daily feed intake.

ADG - average daily gain over the assessed feeding period.

BW - body weight.

TBG - total body weight gain (Kg) during the assessed feeding period.

ABF - average of back fat thicknesses (mm) measured at three points between 6^th^ and 7^th^ ribs (6^th^–7^th^ BF) and at the10th rib (10^th^ BF).

LMA - loin muscle area (cm^2^) measured between the 10^th^ and 11^th^.

IMF - intramuscular fat, percentage IMF determined by the petroleum ether extraction method.

p-value as calculated by t-test.

**Table 2:** List of 20 muscle differentially expressed genes (DEGs) between Yorkshire pigs with low and high RFI.

Gene	RFI_H	RFI_L	FC(L/H)	q_value	Full Name
PPARGC1	65.89	8.63	−7.63	1.52E–02	−
NOR-1	6.83	1.20	−5.68	1.52E−02	−
Unknow1	5.00	0.88	−5.66	1.52E−02	−
HK2	23.88	4.99	−4.78	1.52E−02	hexokinase 2
NTN1	11.01	2.39	−4.61	1.52E−02	netrin 1
CST6	23.82	5.88	−4.05	1.52E−02	cystatin E/M
SYNJ2	4.93	1.26	−3.92	1.52E−02	synaptojanin 2
ESRRB	8.47	2.39	−3.54	1.52E−02	estrogen-related receptor beta
Unknow2	69.28	20.22	−3.43	1.52E−02	−
PON3	16.27	4.92	−3.31	1.52E−02	paraoxonase 3
MYH6	7.22	29.23	4.05	1.52E−02	myosin, heavy chain 6, cardiac muscle, alpha
Unknow12	0.91	3.73	4.12	1.52E−02	−
CD1D	0.66	2.92	4.4	4.55E−02	CD1d molecule
Unknow13	1.75	8.22	4.71	1.52E−02	−
ITGA5	11.20	64.46	5.75	1.52E−02	integrin, alpha 5 (fibronectin receptor, alpha polypeptide)
GGA1	24.08	179.54	7.46	1.52E−02	golgi-associated, gamma adaptin ear containing, ARF binding protein 1
Unknow14	1.14	9.05	7.93	1.52E−02	−
Unknow15	6713.48	55068.8	8.2	1.52E−02	−
CYP1A1	0.97	8.18	8.45	1.52E−02	cytochrome P450, family 1, subfamily A, polypeptide 1
Unknow16	5.41	146.35	27.03	1.52E−02	−

**Table 3:** List of 25 DE miRNAs in LD muscle between Yorkshire pigs with low and high RFI.

Ssc miRNA	Ref miRNA	FC(L/H)	p-value	q-value	Mature Sequence
ssc-miR-215-5p	hsa-miR-215-5p	−673.96	4.57E-27	1.48E-24	augaccuaugaauugacagaca
ssc-new-1	hsa-miR-141-3p	−23.34	2.12E-04	1.73E-02	uaacacugucugguaaagaug
ssc-miR-194-5p	hsa-miR-194-5p	−19.66	8.87E-09	1.44E-06	uguaacagcgacuccauguggac
ssc-miR-365-3p	hsa-miR-365a-3p	−1.94	0.09	1	uaaugccccuaaaaauccuuau
ssc-new-2	hsa-miR-1249	−1.74	0.41	1	acgcccuucccccccuucuuca
ssc-miR-486-5p	hsa-miR-486-5p	−1.69	0.04	1	uccuguacugagcugccccgag
ssc-miR-1271-5p	hsa-miR-1271-5p	−1.64	0.13	1	cuuggcaccuaguaagcacuca
ssc-miR-145-5p	hsa-miR-145-5p	−1.6	0.18	1	guccaguuuucccaggaaucccu
ssc-miR-99b-5p	hsa-miR-99b-5p	−1.51	0.17	1	cacccguagaaccgaccuugcg
ssc-miR-191-5p	hsa-miR-191-5p	−1.5	0.15	1	caacggaaucccaaaagcagcug
ssc-miR-10b-5p	hsa-miR-10b-5p	−1.45	0.14	1	uacccuguagaaccgaauuugu
ssc-miR-29c-3p	hsa-miR-29c-3p	1.48	0.35	1	uagcaccauuugaaaucgguua
ssc-miR-92b-3p	hsa-miR-92b-3p	1.5	0.44	1	uauugcacucgucccggccucc
ssc-miR-101-3p	hsa-miR-101-3p	1.55	0.19	1	uacaguacugugauaacugaag
ssc-miR-338-3p	hsa-miR-338-3p	1.56	0.37	1	uccagcaucagugauuuuguu
ssc-miR-199b-3p	hsa-miR-199b-3p	1.61	0.15	1	acaguagucugcacauugguu
ssc-new-3	hsa-miR-130a-3p	1.73	0.25	1	cagugcaauaguauugucaaagc
ssc-miR-208b-3p	hsa-miR-208b-3p	1.74	0.11	1	auaagacgaacaaaagguuugu
ssc-miR-1-3p	hsa-miR-1	1.81	0.09	1	uggaauguaaagaaguauguau
ssc-miR-30e-5p	hsa-miR-30e-5p	1.83	0.04	1	uguaaacauccuugacuggaagcu
ssc-miR-335-3p	hsa-miR-335-3p	1.89	0.28	1	uuuuucauuauugcuccugacc
ssc-miR-136-3p	hsa-miR-136-3p	1.93	0.12	1	caucaucgucucaaaugagucu
ssc-new-4	hsa-miR-144-3p	2.1	0.32	1	uacaguauagaugauguacu
ssc-new-5	hsa-miR-301b	2.15	0.46	1	cagugcaaugauauugucaaagc
ssc-new-6	hsa-miR-190a	2.28	0.34	1	ugauauguuugauauauuagguug

The p-value was calculated by the formula: 
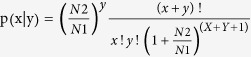
 with a Python script.

The q-value was calculated by “fdrtool” in R, and FDR (false discovery rate) ≤ 0.05 for DEG significant determination.

**Table 4:** Gene ontologies of DE genes in LD muscles from low and high RFI Yorkshire pigs.

Genes	RFI_L/H high	RFI_L/H low	Term ID	Term name	P-Value
**Genes relating to biosynthetic processes**					
PRKAG2 ATP6V0D2 ATP8A1	1	2	GO:0009260	ribonucleotide biosynthetic process	4.73E-02
			GO:0009152	purine ribonucleotide biosynthetic process	4.26E-02
			GO:0009142	nucleoside triphosphate biosynthetic process	3.32E-02
			GO:0009145	purine nucleoside triphosphate biosynthetic process	3.14E-02
			GO:0009201	ribonucleoside triphosphate biosynthetic process	3.14E-02
			GO:0009206	purine ribonucleoside triphosphate biosynthetic process	3.08E-02
			GO:0006754	ATP biosynthetic process	2.58E-02
GM2A, HK2, MDH1	1	2	GO:0016052	carbohydrate catabolic process	3.75E-02
**Genes relating to cell differentiation**					
NOS1, RCAN1, MYH6	1	2	GO:0051146	striated muscle cell differentiation	2.53E-02
			GO:0042692	muscle cell differentiation	4.53E-02
ESRRB, JUNB	0	2	GO:0001829	trophectodermal cell differentiation	4.40E-02
**Genes relating to lipid metabolism**					
GM2A, LIPG, FABP3, ATP8A1	3	1	GO:0010876	lipid localization	9.61E-03
			GO:0006869	lipid transport	7.73E-03
GM2A, LIPG	2	0	GO:0044242	cellular lipid catabolic process	8.62E-02
**Genes relating to energy metabolism**					
PRKAG2, HK2, ATP6V0D2, GLRX, MDH1	0	5	GO:0006091	generation of precursor metabolites and energy	1.12E-02
LIPG, FABP3, SYNJ2, PIK3R1	0	4	GO:0019637	organophosphate metabolic process	1.84E-02
			GO:0006644	phospholipid metabolic process	1.60E-02
FABP3, SYNJ2, PIK3R1	0	3	GO:0006650	glycerophospholipid metabolic process	4.33E-02
**Genes relating to general metabolism**					
PRKAG2, MYH6, ATP6V0D2, ATP8A1	2	2	GO:0009259	ribonucleotide metabolic process	8.03E-03
			GO:0006163	purine nucleotide metabolic process	1.52E-02
			GO:0009150	purine ribonucleotide metabolic process	6.75E-03
			GO:0009141	nucleoside triphosphate metabolic process	5.85E-03
			GO:0009144	purine nucleoside triphosphate metabolic process	4.80E-03
			GO:0009199	ribonucleoside triphosphate metabolic process	4.37E-03
			GO:0009205	purine ribonucleoside triphosphate metabolic process	4.27E-03
			GO:0046034	ATP metabolic process	3.15E-03

RFI_L/H high: the number of genes expressed higher in LD muscle of low RFI pigs compared to RFI high group.

RFI_L/H low: the number of genes expressed lower in LD muscle of low RFI pigs compared to RFI high group.

**Table 5:** List of miRNAs and their target Genes which were both differentially expressed in LD muscle from low and high RFI pigs.

miRNA/Pathway	Target gene counts	Gene Name	Gene Ensembl id
Adipocytokine signaling pathway (hsa04920)			
hsa-miR-130a-3p	1	PPARGC1A	ENSG00000109819
hsa-miR-30e-5p	1	PPARGC1A	ENSG00000109819
hsa-miR-335-3p	1	PRKAG2	ENSG00000106617
hsa-miR-301b	1	PPARGC1A	ENSG00000109819
Insulin signaling pathway (hsa04910)			
hsa-miR-486-5p	1	PIK3R1	ENSG00000145675
hsa-miR-29c-3p	1	PIK3R1	ENSG00000145675
hsa-miR-130a-3p	1	PPARGC1A	ENSG00000109819
hsa-miR-30e-5p	1	PPARGC1A	ENSG00000109819
hsa-miR-335-3p	2	PIK3R1	ENSG00000145675
		PRKAG2	ENSG00000106617
hsa-miR-301b	1	PPARGC1A	ENSG00000109819
Hypertrophic cardiomyopathy (HCM) (hsa05410)			
hsa-miR-92b-3p	1	ITGA5	ENSG00000161638
hsa-miR-335-3p	1	PRKAG2	ENSG00000106617
Bacterial invasion of epithelial cells (hsa05100)			
hsa-miR-486-5p	1	PIK3R1	ENSG00000145675
hsa-miR-29c-3p	1	PIK3R1	ENSG00000145675
hsa-miR-92b-3p	1	ITGA5	ENSG00000161638
hsa-miR-335-3p	1	PIK3R1	ENSG00000145675
Viral carcinogenesis (hsa05203)			
hsa-miR-141-3p	1	ATP6V0D2	ENSG00000147614
hsa-miR-486-5p	1	PIK3R1	ENSG00000145675
hsa-miR-29c-3p	1	PIK3R1	ENSG00000145675
hsa-miR-335-3p	1	PIK3R1	ENSG00000145675
Phagosome (hsa04145)			
hsa-miR-141-3p	1	ATP6V0D2	ENSG00000147614
hsa-miR-92b-3p	1	ITGA5	ENSG00000161638
