# Supplementary material for: Transcriptome analysis of mRNA and miRNA in skeletal muscle indicates an important network for differential Residual Feed Intake in pigs
Source: Sci Rep. 2015 Jul 7;5:11953. doi: 10.1038/srep11953 (PMC4493709; doi:10.1038/srep11953)
Supplement: Supplementary Information [file srep11953-s1.pdf]

Supplementary Information

Title

Transcriptome analysis of mRNA and miRNA in skeletal muscle indicates an important network for differential Residual Feed Intake in pigs

Authors

Lu Jing, Ye Hou, Hui Wu, Yuanxin Miao, Xinyun Li, Jianhua Cao, John Michael Brameld, Tim Parr, Shuhong Zhao*
* the corresponding author

Supplementary Figures

Figure S1 Characterization of RNA-seq mapped reads and mitochondria located genes in longissimus dorsi（LD）muscle from high and low RFI pigs.

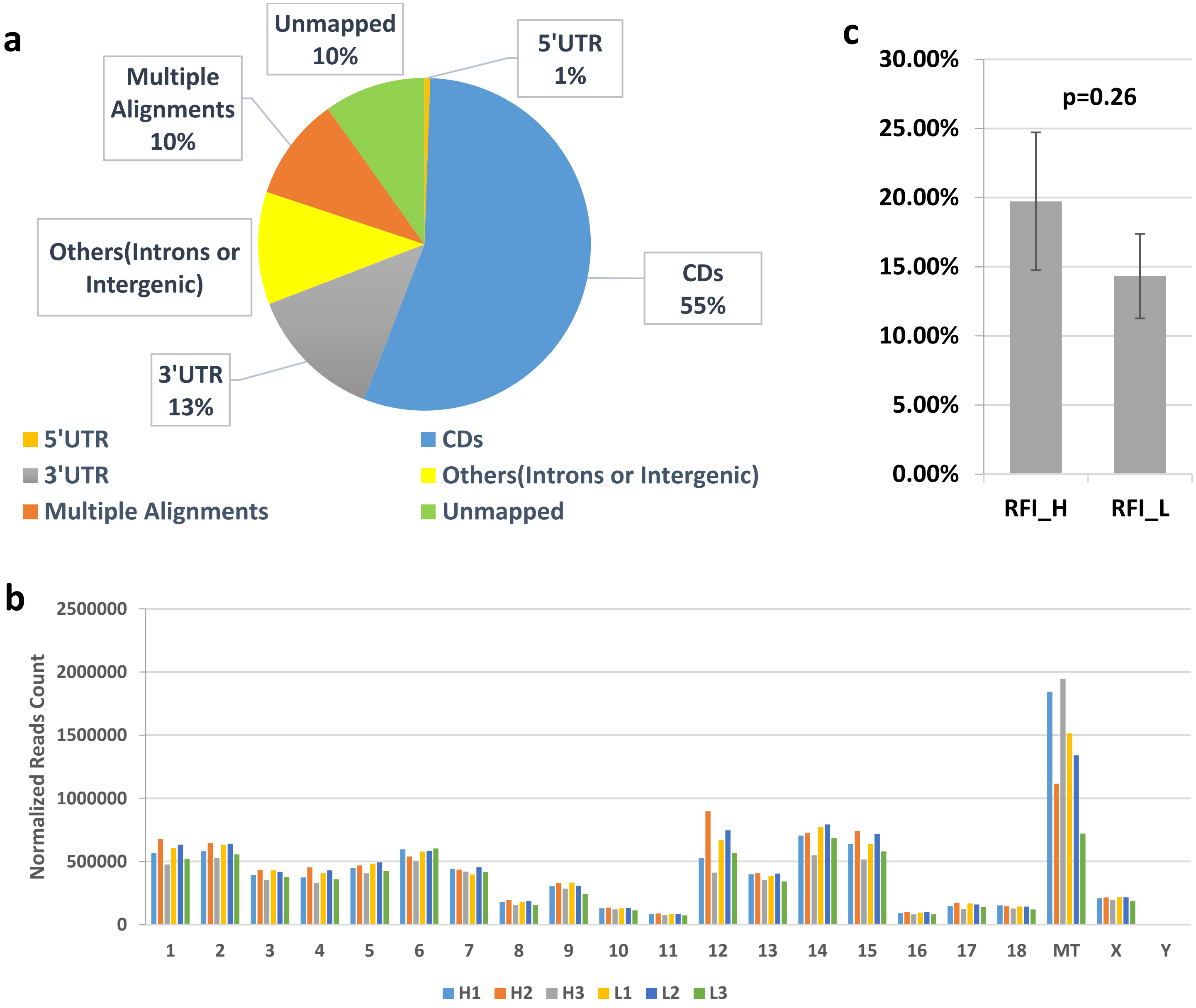

a. Region distribution of mapped reads. Almost 69% of reads were mapped to exon regions.

b. Reads distribution of each chromosome. Each library was normalized by the number of reads mapped to the pig genome. Normalized reads count = number of reads map to chromosome × 1,000,000/ number of reads mapped to genome. H1, H2 and H3 are the 3 RFI_H pigs, while L1, L2 and L3 are the 3 RFI_L pigs.

c. Percentage of reads mapped to mitochondrial DNA..

Figure S2 Characterization of miRNA-seq mapped reads from the miRNA sequencing of porcine LD muscles.

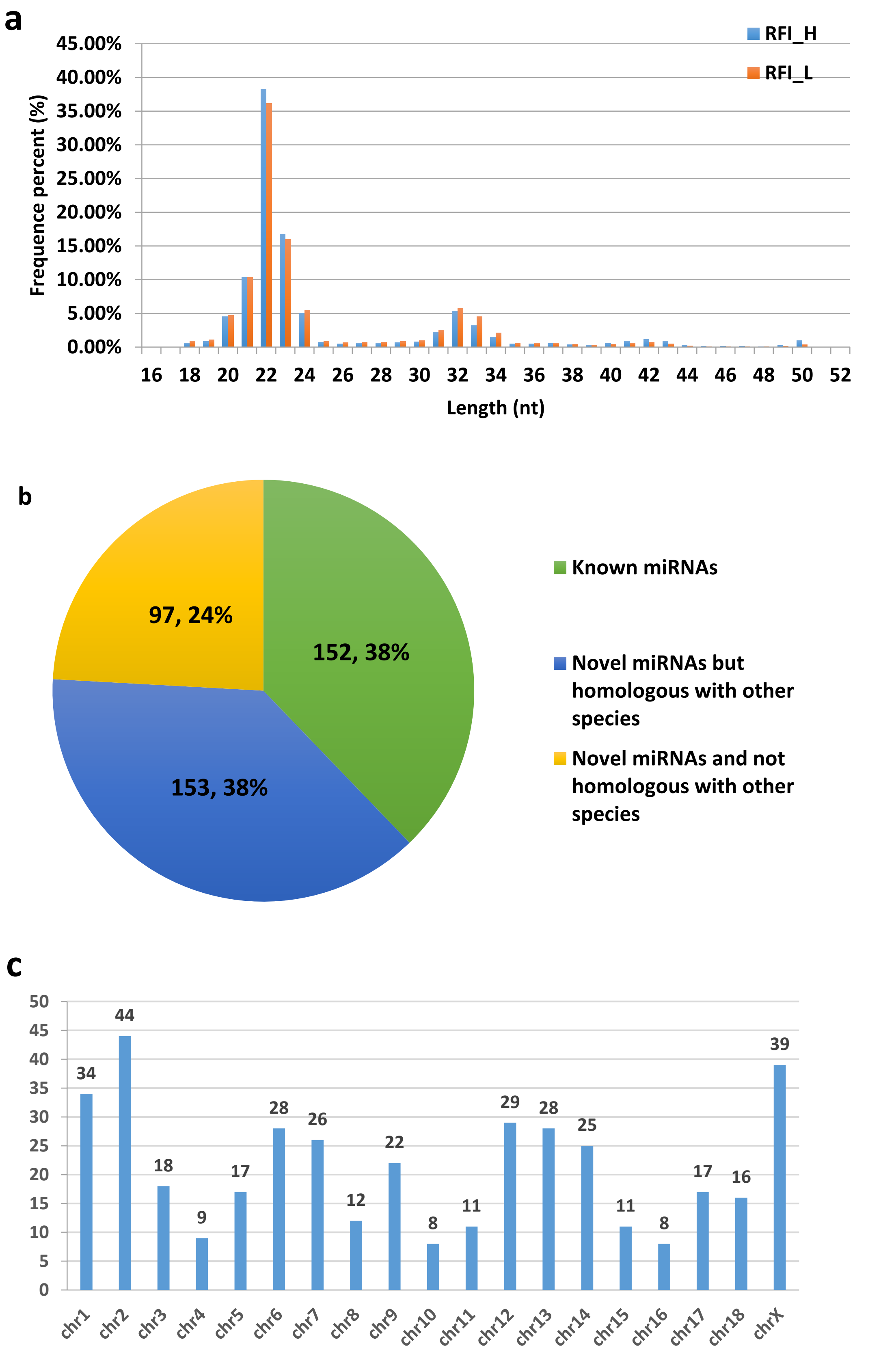

a. The length distribution of clean reads. Frequency percentage = reads count / total reads number of library. most of the reads had a length of 21-23 nt.

b. The numbers and percentage of miRNAs identified. The total was 402 miRNAs.

c. The number of miRNAs found on each chromosome.

Figure S3 Predicted secondary structures of 6 novel porcine muscle miRNAs.

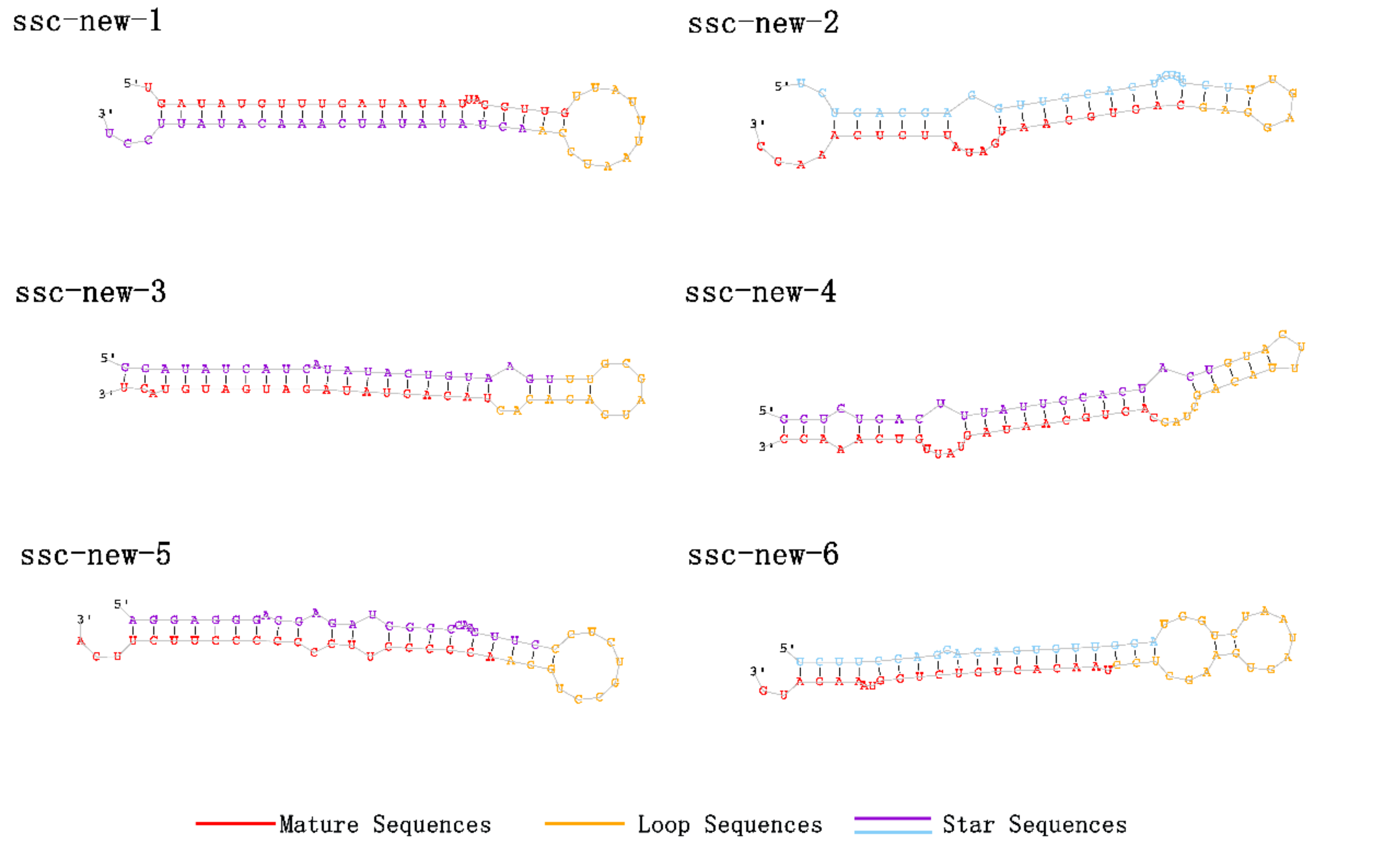

The mature sequences are shown in red, the loop sequences are shown in yellow, the predicted star sequences are shown in blue, and the miRNA star sequences are shown in purple.
